# The unmet need for certification of vision impairment for people accessing a national primary care‐based low vision rehabilitation service

**DOI:** 10.1111/opo.13413

**Published:** 2024-11-01

**Authors:** Rebecca John, Gwyn Williams, Tim Morgan, Michael R. George, Rhianon Reynolds, Jennifer H. Acton

**Affiliations:** ^1^ Wales General Ophthalmic Services, NHS Wales Shared Services Partnership Cardiff UK; ^2^ Singleton Hospital Swansea UK; ^3^ Royal Gwent Hospital Newport UK; ^4^ School of Optometry and Vision Sciences, College of Biomedical and Life Sciences Cardiff University Cardiff UK

**Keywords:** age‐related macular degeneration, certificate of vision impairment, epidemiology, low vision

## Abstract

**Background:**

The certificate of vision impairment has an important role in enabling access to support for people with vision impairment (VI) and the provision of epidemiological data regarding sight loss. However, the rates of certification may not accurately reflect the number of people living with certifiable VI.

**Methods:**

Observational data from a national primary care low vision rehabilitation service between 1 April 2021 and 31 March 2022 were analysed. Descriptive statistics were used to describe the certification status of patients with certifiable VI. For patients with age‐related macular degeneration (AMD) and best‐corrected visual acuity of 6/60 or worse, logistic regression was undertaken to assess the effects of patient characteristics on certification status.

**Results:**

For patients with AMD and certifiable levels of visual acuity, 41.00% (*n* = 426) were not certified. The reported certification was 60.09% (*n* = 256) and 58.24% (*n* = 357) for neovascular AMD and atrophic AMD, respectively. Existing patients of the service were 3.87 times more likely to be certified than new patients (OR 3.87, 95% CI 2.7–5.4). Increasing age (OR 1.02, 95% CI 1.004–1.038) and decreasing visual acuity (OR 0.62, 95% CI 0.50–0.78) were associated with an increased likelihood of certification.

**Conclusion:**

A significant number of patients live with certifiable vision impairment but do not access certification. Policy changes in Wales now enable patients with bilateral atrophic AMD to access certification within the primary care setting. Given the unmet need, consideration should be given to primary care certification in the rest of the UK, and in Wales, the potential to expand the scope of conditions.

**Key points**

- The rates of certification of vision impairment do not reflect the number of people living with certifiable vision impairment.
- This study adds previously unreported population level data regarding the unmet need for certification.
- Given this unmet need, consideration should be given to primary care certification in the rest of the UK, and in Wales, the potential to expand the scope of conditions.

## INTRODUCTION

Certification of vision impairment (CVI) is the prerequisite to formal registration as a person with sight loss with the local government social services department in the UK. Recent changes in June 2023, based on evidence showing comparative agreement between ophthalmologists and optometrists in the identification of eligible patients for CVI,^1^ resulted in a change of Welsh Government policy.^2^ This stated that, in addition to consultant ophthalmologists, optometrists with relevant qualifications are able to certify patients with vision impairment (VI), where the cause is bilateral atrophic age‐related macular degeneration (aAMD).^3^

Historically in the UK, the Blind Person's Act of 1920 introduced a formal register and associated benefits for those living with VI, with any medical practitioner able to certify a patient with a VI. In 1930, the ‘BD8’ was introduced, later replaced by the certificate of vision impairment (CVI) in 2005, and in Wales, known as the CVIW. Timeliness of certification for people with VI was highlighted in the 2019 quality standard (QS180) of the National Institute for Health and Care Excellence,^4^ which states that people should be given a CVI as soon as they become eligible.

Following certification, patients can choose whether to be registered with the local government social services department, which then ensures access to services and support. Certification also allows for collection of epidemiological information regarding the causes and incidence of certifiable sight loss in the UK.^5^, ^6^, ^7^ This information has been used to measure success or otherwise of eye care services and indicates the requirement for additional services, such as specialist habilitative and rehabilitative services. In England, the CVI is used as an indicator of preventable vision impairment as part of the Department of Health Public Health Outcomes Framework.^8^ In Wales, the CVIW is used to report upon the causes and number of people living with certifiable vision impairment.^9^ In Northern Ireland, data are reported at regional clinical audit meetings held at the Belfast Health and Social Care Trust (BHSCT).^7^

However, research has suggested that the number of CVIs does not accurately reflect the number of people living with vision impairment in the UK.^7^, ^10^, ^11^, ^12^, ^13^, ^14^, ^15^, ^16^ There is a need to understand the unmet need for certification in Wales, to assess and inform health and social care service development and delivery.

The primary aim of this study was to assess the unmet need for certification for a population accessing a National Health Service (NHS) low vision rehabilitative service. The secondary aim was to ascertain the factors which may impact upon a person's certification status for patients living with AMD as the cause of VI.

## METHODS

### Data source: Low Vision Service Wales

The Low Vision Service Wales (LVSW) is a primary care NHS low vision rehabilitative service, aimed at maintaining independence of people with VI through the provision of low vision aids, advice, referral and sign posting to other agencies inclusive of social care and the third sector. The LVSW is provided by 200 optometrists and dispensing opticians, who hold the College of Optometrists professional certificate in low vision and have undertaken additional compulsory training provided by Health Education Improvement Wales. The LVSW represents the entirety of optometric low vision services in Wales and has successfully replaced secondary care low vision services in all hospitals.

**FIGURE 1:**
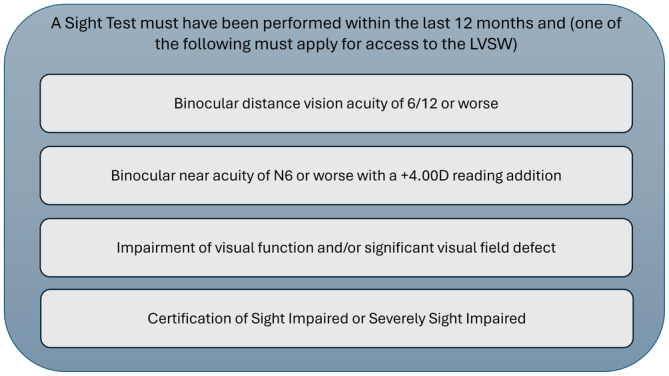
Clinical eligibility criteria for access to the Low Vision Service Wales.

To access the LVSW, the clinical eligibility criteria stated in Figure 1 must be met.^3^

**FIGURE 2:**
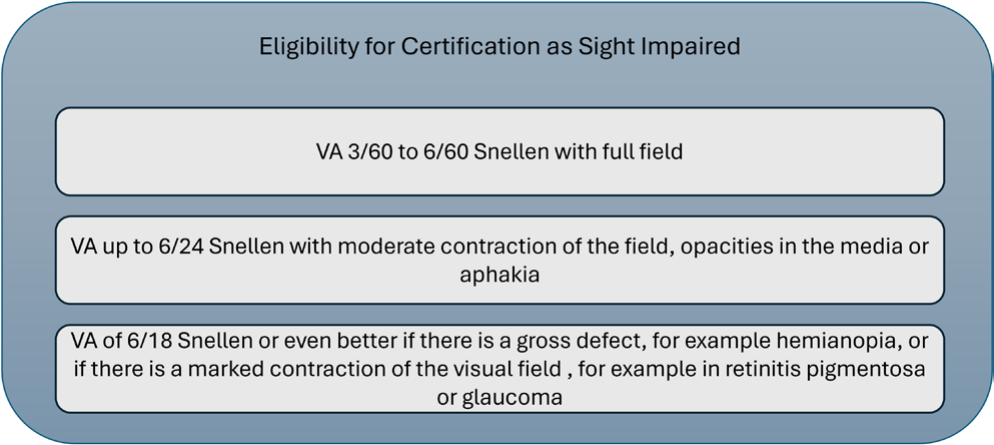
UK guidelines of eligibility for certification as sight impaired. Consideration should also be given to whether the patient has a hearing impairment, time since onset of vision impairment and the patient's social situation.^2^ VA, visual acuity.

Individuals can access the service prior to their visual acuity deteriorating to the level required for the certification of vision impairment. As part of the LVSW assessment, patients' self‐report their certification status as unknown, not certified, sight impaired (SI) (Figure 2) or severely sight impaired (SSI). These criteria are UK wide.

Following each LVSW assessment, the LVSW record^17^ is submitted to NHS Wales Shared Services Partnership (NWSSP) where the data are input into a national database. This facilitates ordering of LVAs, payment for assessment and evaluation of data to inform service delivery.

### Inclusion criteria

Data from records of those patients who had attended for a LVSW assessment between 1 April 2021 and 31 March 2022 were included in the study. Inclusion criteria for analysis of eligibility for certification was a best corrected visual acuity (BCVA)The of 6/60 (1.00 logMAR) or worse. Those aged under 18 years were excluded from the study.

### Analysis

Descriptive statistics and chi‐square significance analyses were conducted using the statistical software package SPSS Version 27 (ibm.com). Binominal regression analysis was performed to assess the effects of age, sex, patient status base (new or existing), type of AMD, presence of a hearing impairment and visual acuity on certification status. Those with atrophic AMD only were categorised as aAMD, those with either neovascular AMD only or both atrophic and neovascular AMD were categorised as nAMD. Significance was reported to a significance value (*α*) = 0.05.

### Patient and Public Involvement

Patients and public were involved in the design of the study via the Low Vision Service Wales Advisory group. The group consists of members who have lived experience of VI. The study concept was explained, and their opinion was sought on the remit, aims and inclusion and exclusion criteria. The ability for patients to self‐report certification status was discussed. The group felt that the accuracy of self‐reporting of certification status was sufficient for study purposes.

Results of the study will be shared with this group and with Wales Vision Forum and Wales Council for the Blind for dissemination to their members on publication.

### Ethics

This study was reviewed by the local ethics committee. It was deemed that no ethical approval was required because the study used routinely collected, de‐identified and anonymised patient data and no patient consent was required. All data were housed and analysed at NWSSP.

## RESULTS

### Demographics

**FIGURE 3:**
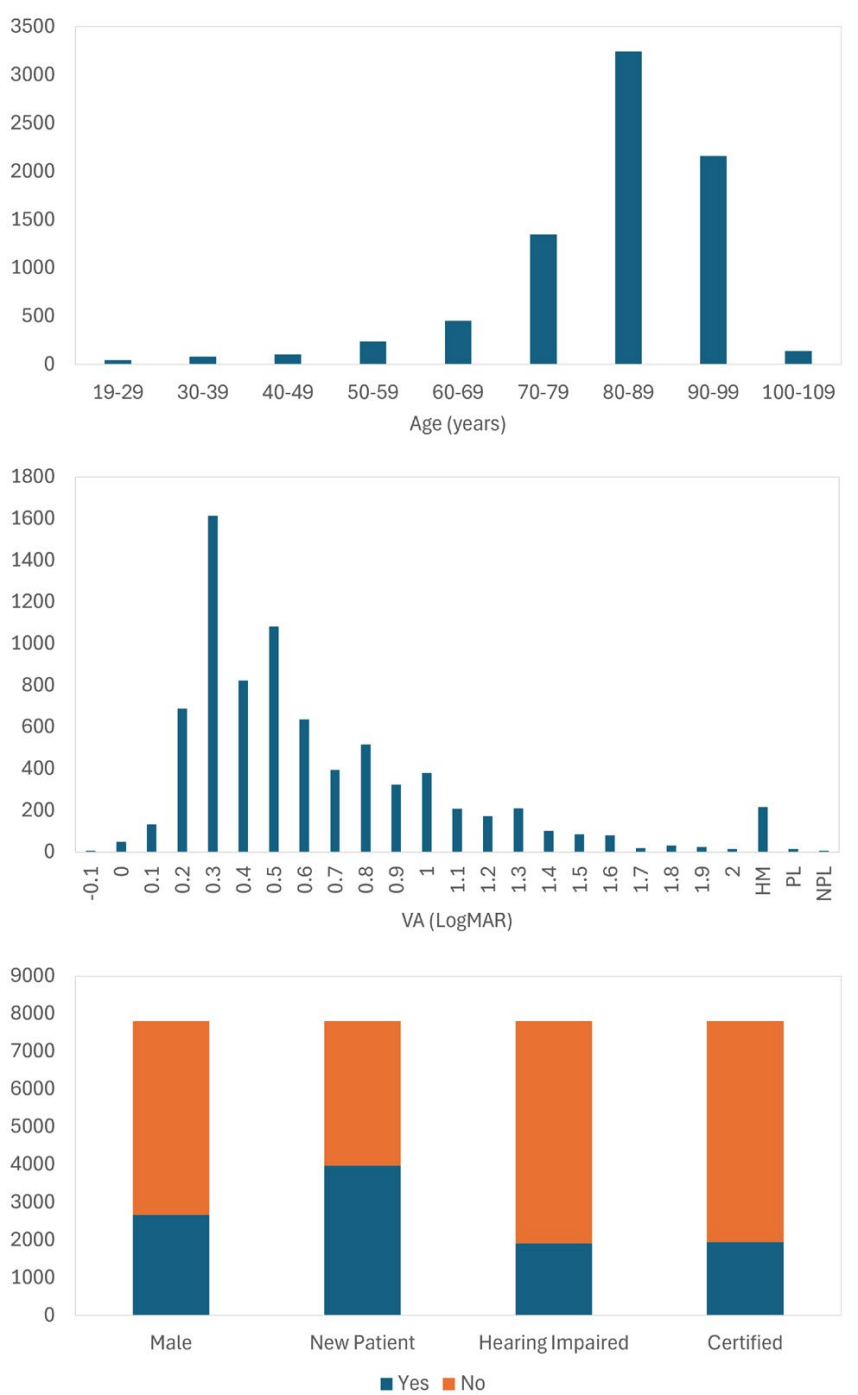
Demographic characteristics of patients attending the Low Vision Service Wales. The top and middle panels show the age distribution and visual acuity of the patients, respectively. The y‐axis in each figure indicates the number of patients. The bottom panel shows the sex, the number of patients who were new to the service, who had a hearing impairment and who were formally certified with a vision impairment.

A total of 7810 adult patients underwent a full LVSW assessment. Of those, 51.88% (*n* = 3974) were new to the service and 49.12% (*n* = 3836) were existing patients, 65.95% (*n* = 5151) were female and 35.05% (*n* = 2659) were male. Overall, hearing impairment was reported by 24.48% (*n* = 1912) and ‘white Caucasian’ was reported as ethnicity by 98.37% (*n* = 7683). Of those who attended, 70.95% (*n* = 5541) were aged 80 years or over (Figure 3).

### Overall certification status: All patients

Of the 7810 patients, 24.94% (*n* = 1948) reported being certified (Figure 3). Of these, 56.37% (*n* = 1098) and 43.63% (*n* = 850) reported being certified as SI and SSI, respectively.

Existing patients of the service were more likely to report being certified than new patients to the service χ^2^ (1, *N* = 7810) = 1039.13, *p* ≤ 0.001, and females more likely to report being certified than males χ^2^ (1, *N* = 7810) = 19.03, *p* ≤ 0.001.

### Overall certification: All causes of VI with a BCVA of 6/60 or worse

For all causes of VI, 19.89% (*n* = 1554) of patients had a BCVA of equal to or worse than 6/60 (1.00 LogMAR), (male 34.49% *n* = 536, female 65.50%, *n* = 1018). Of those, 39.04% (*n* = 607) reported not being certified. Of those with a BCVA of 6/60 or worse, the statistical difference between patient sex (males or female) and reported certification did not persist, χ^2^ (1, *N* = 1554) = 0.834, *p* = 0.36. Where a certifiable level of vision impairment existed, females were no more likely than males to be certified. However, the statistical difference did persist with patient status (new or existing) and reported certification, χ^2^ (1, 1554) = 146.93, *p* ≤ 0.001.

### Certification: Age‐related macular degeneration with a BCVA of 6/60 or worse

Of the 1554 patients seen within the service with a BCVA of equal to or worse than 6/60 (1.00 LogMAR), 66.86% (*n* = 1039) reported AMD as the cause of vision impairment. Of those, 41.00% (*n* = 426) had neovascular (nAMD) or combined nAMD and atrophic AMD (aAMD). The remaining 59.00% (*n* = 613) had aAMD alone (Table 1).

**TABLE 1:** The number and percentage of those with age‐related macular degeneration (AMD) with a best‐corrected visual acuity of 6/60 or worse and certified into subgroups of neovascular (nAMD) or combined and atrophic AMD (aAMD).

Condition	Certification status				
	Certified yes		Certified no		Total
	*n*	%	*n*	%	
nAMD	256	60.09	170	39.91	426
aAMD	357	58.24	256	41.74	613
Total AMD (all)	613	59.00	426	41.00	1039

The reported certification of those with a BCVA of equal to or less than 6/60 (1.00 LogMAR), was 60.09% (*n* = 256) and 58.24% (*n* = 357) for nAMD and aAMD, respectively. Those with nAMD were just as likely to be certified as those with aAMD, χ^2^ (1, *N* = 1039) = 0.36, *p* = 0.55.

Overall, for all types of AMD, 59.00% of patients eligible for certification reported being certified; therefore, 41% (*n* = 426) of patients with a BVCA of 6/60 or worse (1.00 LogMAR) were not certified (Table 1).

A binominal logistic regression was performed to ascertain the effects of age, sex, patient status (new or existing), type of AMD (nAMD or aAMD), the presence of a hearing impairment and visual acuity on the likelihood that patients were certified. The logistic regression model was statistically significant, *X* ^2^(6) = 90.26, *p* < 0.0005. The model explained 11.2% (Nagelkerke *R* ^2^) of the variance in certification status and correctly classified 65.3% of cases (sensitivity 29.3%, specificity 90.4%) (Table 2).

**TABLE 2:** Classification table. The cutoff was 0.50. The model explained 63.5% of cases correctly (sensitivity 29.3% and specificity 90.4%).

Observed	Predicted		
	Certified		
	yes	no	Percentage correct
Certified yes	553	59	90.4
Certified no	301	125	29.3
Overall percentage			65.3

Existing patients of the service were 3.87 times more likely to be certified than new patients (OR 3.87, 95% CI 2.7–5.4). Increasing age (OR 1.02, 95% CI 1.00–1.04) and decreasing visual acuity (OR 0.62, 95% CI 0.50–0.78) were associated with an increased likelihood of certification. Patient sex, the presence of a hearing impairment and the type of AMD were not related to certification status (Table 3). The model exhibited a good fit with an area under the curve (AUC) of 0.66.

**TABLE 3:** Logistic regression results. Base categories were, sex: Female, new patient: Yes, type of age‐related macular degeneration (AMD): Neovascular; hearing impairment: Yes.

Variable	*B*	SE	Wald	df	Significance	Exp(B)	95% CI for EXP(B)	
							Lower	Upper
Age	0.02	0.009	6.01	1	0.01	1.02	1.00	1.04
Sex	0.12	0.14	0.66	1	0.42	1.12	0.85	1.49
New patient	1.35	0.18	59.76	1	<0.001	3.87	2.74	5.45
Type of AMD	−0.15	0.14	1.24	1	0.27	0.86	0.66	1.12
Hearing impairment	−0.20	0.15	1.77	1	0.18	0.82	0.61	1.10
Visual acuity (LogMAR)	−0.47	0.11	17.28	1	<0.001	0.62	0.50	0.78
Constant	−1.73	0.76	5.17	1	0.02	0.18		

Abbreviations: CI, confidence intervals; df, degrees of freedom; SE, standard error.

## DISCUSSION

### Key findings

This study reports upon the certification status of patients accessing a national primary care‐based low vision rehabilitation service immediately prior to a CVI policy change in Wales, in which optometrists with relevant qualifications can certify patients with bilateral aAMD with VI. The results suggest that overall, around 40% of those who access the LVSW and have a certifiable level of visual acuity are not certified. Furthermore, only 19.89% of patients accessing the service have certifiable VI defined as a BCVA of equal to or worse than 6/60 (1.00 LogMAR). This supports that certifiable VI may be a poor indicator of whether a patient requires rehabilitative support^16^ and highlights that the LVSW is an essential service both for those who do not yet have certifiable VI, and for those who, despite being certifiable as sight impaired, are not certified.

Individuals with nAMD were no more likely to be certified than those with aAMD. However, existing patients to the service were more likely to be certified than new patients, as were individuals with lower VA and of older age. These results support the previous findings that the numbers of CVI in the UK do not reflect the numbers of people living with certifiable sight loss.^10^, ^11^, ^12^, ^13^, ^14^, ^15^

Primarily, the findings provide an evidence base for the certification status of people accessing the LVSW and highlight the need for an increase in certification for this vulnerable population. They allow estimation of the immediate expected increase in CVIW for those with aAMD, who since June 2023 can be certified by optometrists within the LVSW.^2^ Additionally, the results emphasise the increase in demand for services such as specialist rehabilitation and provision of financial benefits,^18^ which could be expected if all those eligible were to access certification.

It has previously been postulated that a potential barrier to certification is the necessity of a referral into the secondary care setting.^13^, ^19^ While this may be the case, the present findings also highlight that those with nAMD who are accustomed to accessing the hospital eye service on a frequent basis, are not necessarily accessing the certification process. The reasons for this are likely to be complex and may be related to time available within busy clinics,^14^, ^20^ geographical variation,^12^ consultant ophthalmologist variation in practice,^20^ the consideration that nAMD is treatable^11^, ^21^, ^22^ and understanding of the certification process and associated benefits.^23^ Therefore, it cannot be assumed that patients accessing secondary care clinics are able to readily access timely certification.^15^

It is expected that many of these barriers will be alleviated with the introduction of certification within the primary care setting. For example, the development of professional guidance^3^ was introduced alongside dedicated clinic time for certification. Another benefit is the increased number of clinicians able to undertake certification in Wales. Qualitative studies performed with patients with vision impairment highlight patient confusion around the certification process and the need for more information being provided to patients regarding both the process and the associated benefits.^19^, ^23^ Efforts have been made to address these issues within the primary care certification pathway, for example, through the development of new patient information regarding the process and benefits of certification.^2^

However, it is possible that some barriers will persist. Although there is a paucity of research into the psychosocial impact of certification, it is known that VI increases a person's risk of depression,^24^, ^25^, ^26^ social isolation^27^ and loneliness.^28^, ^29^ It is plausible that there may be psychosocial barriers to patients accepting the offer of certification.

### Strengths and limitations

The key strength of this study is the large data set and the use of national population level data.

A limitation of the study is the integrity of the data as the determination of certification status relied upon patient self‐reporting. In line with the UK national guidance on certification,^2^ it is acknowledged that patients may be certified when BCVA is better than 6/60 (1.00 LogMAR); for example in those with visual field loss or lens opacities. It was beyond the granularity of the data available to ascertain the definitive eligibility criteria for each patient seen within the LVSW.

A further limitation is the reported data relates to those patients who are actively accessing eye care services. It is likely that there are people living with certifiable VI who are not accessing such services. Therefore, the results present the ‘best case scenario’ regarding the numbers of people living with certifiable VI but not accessing certification in Wales.

## CONCLUSION

A significant number of patients live with certifiable vision impairment but do not access certification. Policy changes in Wales now enable patients with bilateral aAMD to access certification within the primary care setting. Given the unmet need, consideration should be given to primary care certification in other countries, and in Wales, the potential to expand the scope of conditions.

The development to the certification pathway in Wales provides precedent for the development of formal recording of VI in other countries where there is a limitation of access due to a restricted number of professionals able to perform certification.

Until the number of certifications more accurately aligns with the number of people living with certifiable VI, using the CVI as a measure of those living with certifiable VI should be approached with caution. Furthermore, the number of CVIs should not be used as an outcome measure for vision services.

## AUTHOR CONTRIBUTIONS

**Rebecca John:** Conceptualization (lead); data curation (lead); formal analysis (lead); investigation (lead); methodology (lead); project administration (lead); resources (lead); validation (lead); visualization (lead); writing – original draft (lead); writing – review and editing (lead). **Gwyn Williams:** Conceptualization (supporting); writing – original draft (supporting); writing – review and editing (supporting). **Tim Morgan:** Conceptualization (supporting); investigation (supporting); methodology (supporting); supervision (supporting); visualization (supporting); writing – original draft (supporting); writing – review and editing (supporting). **Michael R. George:** Conceptualization (supporting); investigation (supporting); methodology (supporting); supervision (supporting); visualization (supporting); writing – original draft (supporting); writing – review and editing (supporting). **Rhianon Reynolds:** Writing – original draft (supporting); writing – review and editing (supporting). **Jennifer H. Acton:** Formal analysis (supporting); methodology (supporting); supervision (lead); validation (supporting); visualization (supporting); writing – original draft (supporting); writing – review and editing (supporting).

## FUNDING INFORMATION

his study did not recieve any funding.

## CONFLICT OF INTEREST STATEMENT

The authors declare that they have no conflict of interest.
